# Open Approach to Primary Lumbar Hernia Repair: A Lucid Option

**DOI:** 10.1155/2017/5839491

**Published:** 2017-10-17

**Authors:** Ketan Vagholkar, Suvarna Vagholkar

**Affiliations:** Department of Surgery, D. Y. Patil University School of Medicine, Nerul, Navi Mumbai 400706, India

## Abstract

**Background:**

Lumbar hernia is a rare type of hernia. Awareness of the anatomical basis of this hernia is important for proper diagnosis and treatment.

**Introduction:**

Lumbar hernia is a protrusion of either extraperitoneal fat or intraperitoneal contents through either of the lumbar triangles. Primary lumbar hernias are extremely rare thereby rendering such a case reportable, to create an awareness about this condition to upcoming surgeons.

**Case Report:**

A case of primary lumbar hernia treated successfully by open mesh repair is presented.

**Discussion:**

The anatomical aspects underlying this condition along with diagnostic tests, their pitfalls, and surgical approaches are discussed.

**Conclusion:**

Awareness of this condition is essential for arriving at a clinical diagnosis. CT scan provides a road map for deciding the approach. Both the traditional open and the newer laparoscopic approaches are described. However open meshplasty is still a very safe and effective method of treatment.

## 1. Introduction

A lumbar hernia is best defined as a protrusion of either extraperitoneal or intraperitoneal contents through a defect situated in the posterolateral abdominal wall. Barbette was the first to describe this entity in 1672 [1]. Subsequently Petit and Grynfeltt described the anatomical boundaries of the inferior and superior lumbar triangles, respectively [2]. Majority of lumbar hernias arise from these anatomical sites. Due to the rarity of this type of hernia, diagnosis and management of this hernia always pose a challenge to the attending surgeon.

## 2. Case Report

**Figure 1:**
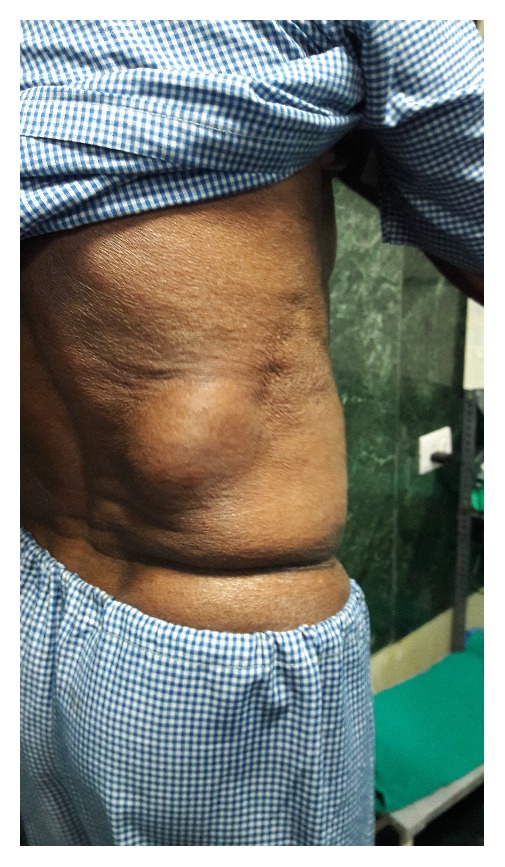
Hernia arising from the lumbar region just below the twelfth rib on the right side.

A 54-year-old male presented with a swelling in the right lumbar region for one year. There was no history of any undue straining or any medical comorbidities. Physical examination revealed a bulge arising below the twelfth rib (Figure 1). The bulge disappeared on lying down and became obvious on standing and coughing. Both a visible and palpable impulse on coughing was appreciated. A contrast enhanced CT scan was done and revealed a defect in the posterolateral body wall with protrusion of omentum.

**Figure 2:**
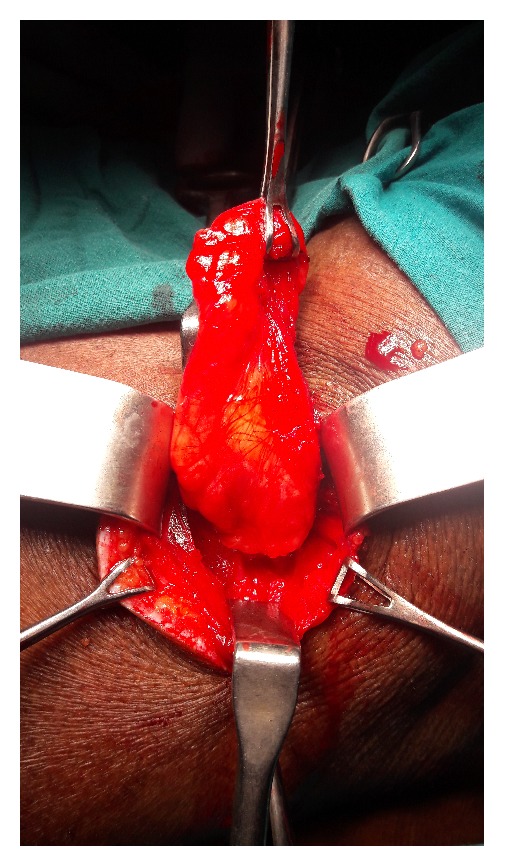
Retroperitoneal fat dissected out up to the defect.

**Figure 3:**
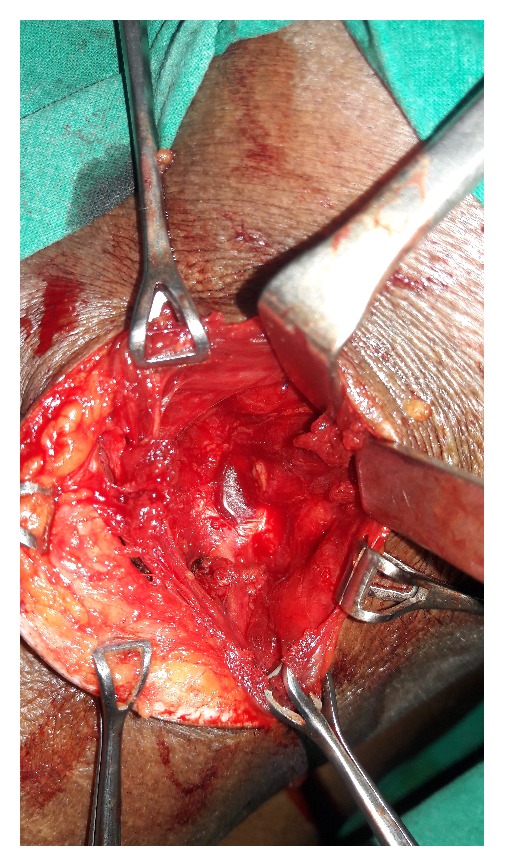
Defect delineated after excision of the protruding retroperitoneal fat.

**Figure 4:**
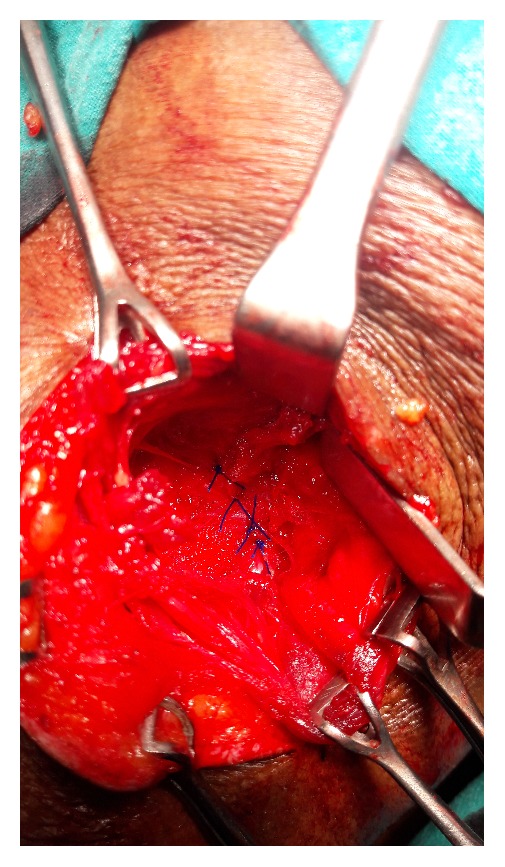
Fascial flaps sutured over the defect.

**Figure 5:**
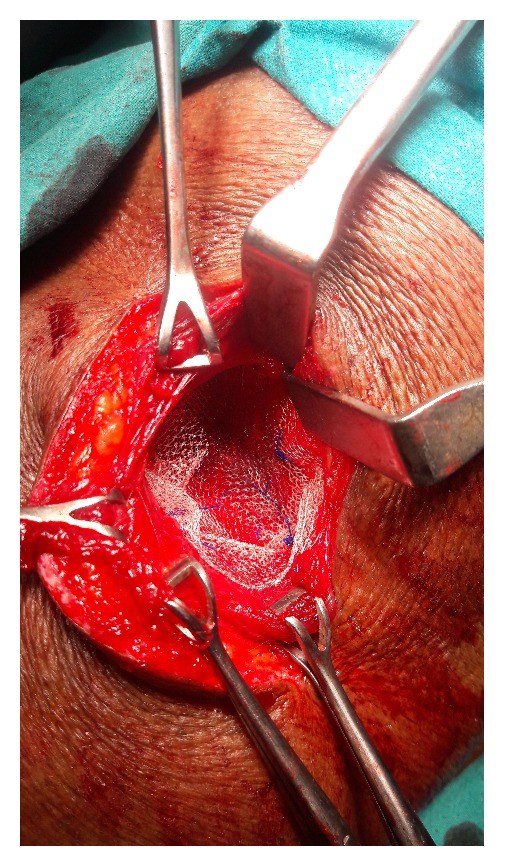
Mesh placed over the sutured fascial flaps and underneath the muscle layer (Sublay).

**Figure 6:**
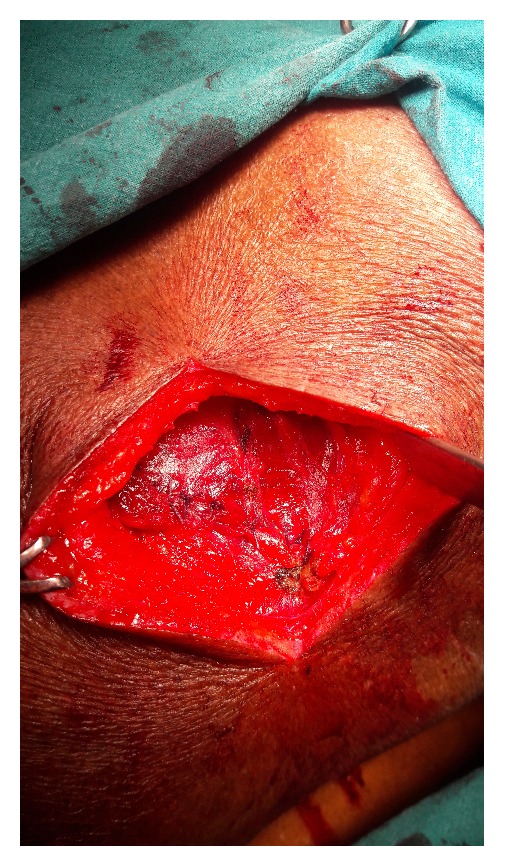
Muscle flaps approximated over the mesh.

An open approach through a posterolateral lumbar incision overlying the swelling was adopted. The retroperitoneal fat constituted the hernia (Figure 2). The content was dissected up to the neck of the sac. The extraperitoneal fat was excised. The defect was clearly appreciated and delineated (Figure 3). The herniation was through the superior lumbar triangle. The loose lumbar fascia adjacent to the defect was dissected enough to create flaps for approximation. The fascia was approximated with interrupted Prolene stitches (Figure 4). The attenuated muscle layers were dissected by undermining thereby creating good muscle flaps. A Prolene mesh was placed over this fascial layer and fixed to the overhanging muscle flaps (Figure 5). The muscle flaps were approximated over the mesh (Figure 6). Care was taken at every step to ensure a tension-free repair. The postoperative recovery was uneventful. The patient has followed up for one year with no recurrence.

## 3. Discussion

The rarity of lumbar hernias renders this condition enigmatic. A surgeon may not even encounter this type of hernia in his entire career as a surgeon. Hence awareness of this distinct entity is pivotal to avoid mismanagement. Understanding the intricate anatomy of the region is essential for a good repair. There are triangles described in this area. The superior lumbar triangle is described by Grynfeltt and the inferior lumbar triangle by Petit. The boundaries of the superior triangle are the posterior border of the internal oblique muscle anteriorly, the anterior border of the sacrospinalis posteriorly, the twelfth rib, and the serratus posterior inferior muscle superiorly. The floor is formed by the aponeurosis of the transversus abdominis and the roof by the external oblique and latissimus dorsi. The inferior lumbar triangle is formed by the external oblique anteriorly, by the anterior border of the latissimus dorsi posteriorly, and below by the iliac crest. The internal oblique constitutes the floor and loose fascia of the roof. Therefore the contents of a hernia arising from any of these triangles could be retroperitoneal fat, kidney, colon, and omentum. There is increased possibility of incarceration in these hernias.

Lumbar hernias can be classified into two types: congenital and acquired. Congenital hernias are very rare and are associated with multiple musculoskeletal anomalies in that region typically described as lumbocostovertebral syndrome [3]. The acquired variety may be either primary or secondary. Primary variety is very rare with approximately 300 cases being described in literature. The acquired variety is usually seen after operations such as iliac bone graft harvesting or drainage of abscesses in that region [4].

The clinical presentation is quite straightforward with a bulge in the lumbar region which exhibits both a visible and palpable impulse on coughing. The bulge will disappear on lying flat in a lateral position and become prominent on standing up and coughing. Ignorance of the existence of this entity may lead to misdiagnosis as a lipoma or an abscess which can have disastrous consequences [5, 6]. Contrast enhanced CT scan is essential before a surgical repair [7, 8]. The only pitfall of CT scan is that retroperitoneal fat is invariably misinterpreted as omentum. However if organs find their way into the sac then these can be identified preoperatively providing a road map for surgical repair [8].

Surgical repair is the mainstay of treatment [9]. The traditional open approach still holds true [10]. With the advent of minimally invasive surgery, laparoscopic approach has gained popularity and is strongly advocated by some [10, 11]. Open repair has evolved over a period of time. Proper delineation of the defect followed by tension-free placement of a sublay mesh yields good results especially in primary lumbar hernias. However in acquired type or secondary type of acquired type of lumbar hernias, advanced muscle flaps may be required over and above the mesh to ensure complete coverage of the defect. Despite the best of surgical repair, failures have been described [4, 11]. They have been attributed to limited fascial strength, weakening of the surrounding musculoaponeurotic structures, inadequate hold of sutures to weakened tissues, and bony edges. Laparoscopic repair may be done by either an extraperitoneal or transperitoneal approach with placement of a mesh. Laparoscopic repair confers certain advantages. Reduced operative morbidity, reduced pain, and early return to routine activity are established advantages [11]. However long term outcomes with respect to morbidity and recurrence rates do not differ [11]. Therefore depending upon the site and size of the defect, contents of the sac, the attenuated state of the surrounding tissues, and cost factor, a tailor made repair has to be performed to ensure successful outcome of surgical intervention.

## 4. Conclusion

Awareness of the anatomy of the lumbar triangles is essential for prompt diagnosis of lumbar hernias.

A contrast enhanced CT scan is essential for confirming the diagnosis.

Repair can be done by both laparoscopic and open approach. Open mesh repair is an easy, safe, and effective means of curing this rare surgical condition.

## References

[1] Sundaramurthy S., Suresh H. B., Anirudh A. V., Prakash Rozario A. (2016). Primary lumbar hernia: A rarely encountered hernia. *International Journal of Surgery Case Reports*.

[2] Sharma P. (2009). Lumbar hernia. *Medical Journal Armed Forces India*.

[3] Vagholkar K., Dastoor K. (2013). Congenital lumbar hernia with lumbocostovertebral syndrome: a case report and review of the literature. *Case Reports in Pediatrics*.

[4] Vagholkar K., Budhkar A., Gulati J. (2014). Lumbar Incisional Hernia Repair following Iliac Bone Graft Harvest. *Journal of Case Reports*.

[5] Mingolla G. P., Amelio G. (2009). Lumbar hernia misdiagnosed as a subcutaneous lipoma: A case report. *Journal of Medical Case Reports*.

[6] Zub A., Kozka M. (2003). Petit’s triangle hernia clinically mimicking gluteal abscess. *Przegl Lek*.

[7] Boker M. E., Wenerth J. L., Andiani R. T. (1987). Lumbar hernia: diagnosis by CT. *American Journal of Roentgenology*.

[8] Guillem P., Czarnecki E., Duval G., Bounoua F., Fontaine C. (2002). Lumbar hernia: anatomical route assessed by computed tomography. *Surgical and Radiologic Anatomy*.

[9] Moreno-Egea A., Baena E. G., Calle M. C., Martínez J. A. T., Albasini J. L. A. (2007). Controversies in the current management of lumbar hernias. *JAMA Surgery*.

[10] Moreno-Egea A., Torralba-Martinez J. A., Morales G., Fernández T., Girela E., Aguayo-Albasini J. L. (2005). Open vs laparoscopic repair of secondary lumbar hernias: a prospective nonrandomized study. *Surgical Endoscopy*.

[11] Moreno-Egea A., Alcaraz A. C., Cuervo M. C. (2013). Surgical options in lumbar hernia: Laparoscopic versus open repair. A long-term prospective study. *Surgical Innovation*.

